# Supplementary material for: Lymphatic filariasis in 2016 in American Samoa: Identifying clustering and hotspots using non-spatial and three spatial analytical methods
Source: PLoS Negl Trop Dis. 2022 Mar 28;16(3):e0010262. doi: 10.1371/journal.pntd.0010262 (PMC8989349; doi:10.1371/journal.pntd.0010262)
Supplement: S1 Table — (DOCX) [file pntd.0010262.s001.docx]

**S1 Table. Number and percentage of households (n=750) with participants with positive infection markers**

| **Number of positive persons per**  **household** | **Antigen** | | **Microfilaria** | | **Wb123 Ab** | | **Bm14 Ab** | | **Bm33 Ab** | |
| --- | --- | --- | --- | --- | --- | --- | --- | --- | --- | --- |
|  | **Number of households** | **%** | **Number of households** | **%** | **Number of households** | **%** | **Number of households** | **%** | **Number of households** | **%** |
| 0 | 658 | 87.7 | 725 | 96.7 | 343 | 45.7 | 506 | 67.5 | 169 | 22.5 |
| 1 | 69 | 9.2 | 21 | 2.8 | 252 | 33.6 | 182 | 24.3 | 259 | 34.5 |
| 2 | 15 | 2.0 | 1 | 0.1 | 88 | 11.7 | 44 | 5.9 | 166 | 22.1 |
| >2 | 8 | 1.1 | 3 | 0.4 | 67 | 8.9 | 18 | 2.4 | 156 | 20.8 |
| At least 1 | 92 | 12.3 | 25 | 3.3 | 407 | 54.3 | 244 | 32.5 | 581 | 77.5 |
