# Supplementary material for: Lymphatic filariasis in 2016 in American Samoa: Identifying clustering and hotspots using non-spatial and three spatial analytical methods
Source: PLoS Negl Trop Dis. 2022 Mar 28;16(3):e0010262. doi: 10.1371/journal.pntd.0010262 (PMC8989349; doi:10.1371/journal.pntd.0010262)
Supplement: S2 Table — (DOCX) [file pntd.0010262.s002.docx]

**S2 Table. Number and percentage of unique survey locations (n=730) with participants with positive infection markers**

| **Number of positive persons per**  **Survey location** | **Antigen** | | **Microfilaria** | | **Wb123 Ab** | | **Bm14 Ab** | | **Bm33 Ab** | |
| --- | --- | --- | --- | --- | --- | --- | --- | --- | --- | --- |
|  | **Number of households** | **%** | **Number of households** | **%** | **Number of households** | **%** | **Number of households** | **%** | **Number of households** | **%** |
| 0 | 639 | 87.5 | 705 | 96.6 | 328 | 44.9 | 487 | 66.7 | 157 | 21.5 |
| 1 | 68 | 9.3 | 21 | 2.9 | 245 | 33.6 | 182 | 24.9 | 254 | 34.8 |
| 2 | 14 | 1.9 | 1 | 0.1 | 90 | 12.3 | 43 | 5.9 | 163 | 22.3 |
| >2 | 9 | 1.3 | 3 | 0.4 | 67 | 9.2 | 18 | 2.5 | 156 | 21.4 |
| At least 1 | 91 | 12.5 | 25 | 3.4 | 402 | 55.1 | 243 | 33.3 | 573 | 78.5 |
