# Supplementary material for: Lymphatic filariasis in 2016 in American Samoa: Identifying clustering and hotspots using non-spatial and three spatial analytical methods
Source: PLoS Negl Trop Dis. 2022 Mar 28;16(3):e0010262. doi: 10.1371/journal.pntd.0010262 (PMC8989349; doi:10.1371/journal.pntd.0010262)
Supplement: S3 Table — (DOCX) [file pntd.0010262.s003.docx]

**S3 Table. Moran’s I statistics**

| **Seromarkers** | **Moran’s Index** | **Variance** | **z-score** | **p-value** |
| --- | --- | --- | --- | --- |
| FTS | 0.06687 | 0.000053 | 9.383436 | <0.0001 |
| MF | 0.045148 | 0.00005 | 6.606337 | <0.0001 |
| Wb123 | 0.067202 | 0.000056 | 9.189932 | <0.0001 |
| Bm14 Ab | 0.099704 | 0.000055 | 13.667150 | <0.0001 |
| Bm33 Ab | 0.039730 | 0.000056 | 5.490717 | <0.0001 |
