# Supplementary material for: Lymphatic filariasis in 2016 in American Samoa: Identifying clustering and hotspots using non-spatial and three spatial analytical methods
Source: PLoS Negl Trop Dis. 2022 Mar 28;16(3):e0010262. doi: 10.1371/journal.pntd.0010262 (PMC8989349; doi:10.1371/journal.pntd.0010262)
Supplement: Attachment — Submitted filename: Comments on PNTD-D-21-00923_reviewer Dr.A.Srividya.docx [file pntd.0010262.s004.docx]

**Comments on the manuscript entitled ”Lymphatic filariasis in 2016 in American Samoa: Identifying clustering and hotspots using non-spatial and three spatial analytical methods” by Wangdi et al**

The manuscript is relevant in the current context where in most endemic countries after several rounds of MDA, the filarial infection in the endemic population have reached very low levels. And even more important in identifying such active transmission foci, which when left unattended may lead to resurgence in the absence of MDA

1. The authors have used 5 infection indicators Mf, Ag, Wb123, Bm14 and Bm33. It would be useful for the reader if they also indicate what each indicator measures, as this has implications later while discussing about their spatial patterns.
   a. How useful is B33 as this is known for its cross reactivity with other parasites?
   b. A table with sitewise details of samples size, Mf, Ag and the different Ab prevalences could be provided so that it would be useful while interpreting the findings of the spatial analysis
2. Line 124-25: The authors say the adjusted Ag prevalence- what do they mean by that? How was that estimated after adjusting for which variables – the authors may explain briefly in few lines
3. Lines 108-113: This is nothing new, as it is expected that the older age classes will have higher infection levels compared to 6-7 yo, due to their prolonged exposure (even prior to the MDAs). But how does one interpret whether these are really due on-going transmission as the Ag and Ab status do remain positive even after repeated rounds of MDA, even when Mf is zero or levels are very low.
4. Lines 234-246: The authors may kindly include a statement that approval was obtained to take blood samples (mention quantity) from the participants in the study.
5. Lines 274-277: Did the authors try fitting any distributions to the frequency distribution of no. of infected persons per household for different indicators? (Like Poisson or Negative binomial). It can be tried as well as this also will give an idea on the distribution pattern of cases for different indicators at household and also at site level
6. Lines: 284-288: As the authors have used mixed effects LR model with households and villages as random effects in their respective models, the Table 1 to include the significance of the ICC estimated for different infection indicators.
   a. What did the authors interpret from the clustering within households or villages? What could be the reasons for such clustering?
   b. A multilevel analysis accounting for village and household will help in assessing the contribution of variation due to different levels – could be carried out so that appropriate interventions could be undertaken at different levels
7. Lines-290-295: The authors give semivariogarms and its parameters of spatial autocorrelation like sill, partial sill, nugget etc. Instead, it is ideal to estimate the spatial autocorrelation (Moran’s I) and also assess its significance before interpreting the parameters like sill, partial sill etc and giving the size of the clusters. This has to be estimated for each of the infection indicators to see if the spatial dependency is really significant.
8. Fig 7 - The semivariograms for Ag and Mf do not show any pattern o spatial dependency compared to the Ab, though it is mentioned there is clustering. A moran’s I would be more useful here to assess.
9. Lines: 296-305:
   a. How was the window size decided for SaTScan tool?
   b. From the results its understood all analysis were at household level ie household geolocations?
   c. Since there were 30 sites, was clustering identified at village level also? If so what were the results.
   d. When more than one cluster is formed, which cluster is more significant and what differs between different clusters, in terms of range of prevalence.
   e. What was the range of observed prevalence of different indicators in the clusters identified by SaTScan? It would be interesting to see how these clusters are. A Table would suffice.
10. Lines 306-316: If correct, Getis-Ord General G tool is most appropriate there is an even distribution of values with spatial spikes of high values. However when both high and low values cluster in space, measuring spatial clustering using spatial autocorrelation is a better tool which also has means to test if those clusters are significant as well.. Since all the infection indicators are showing both low and high clusters, more appropriate tool to study spatial heterogeneity would be estimate the spatial autocorrelation statistics and interpret the patterns based on their significance.
11. Lines 377-381: Authors say in 2016 it’s a resurgence –
    a. How does one define resurgence?
    b. What are the associated cut-off levels for these different indicators?
    c. Authors to kindly explain these with appropriate references.
    d. This contradicts with the statement in lines: 59-60 where they address these as residual hotspots and areas with on-going transmission. The authors should be very clear in what they want to emphasize.
12. Lines: 343-45: The authors should explain why the results varied with valid interpretations in terms of indicators and the methods used by different spatial analytical methods.
13. Lines: 346-47: the authors say “identified significant spatial dependency for all infection markers, with different cluster size for each marker”. Was any significance testing done? Those details are not given in the results. Please provide them
14. Lines: 353-358: A multilevel analysis could help in assessing which level is contributing more to the variation in these infection indicators.
15. Lines: 358-360: Is identifying clusters of positive individuals only for testing and treating them? How about using these information for targeted intervention?
16. Lines: 366-371: With the dynamics of environment both at micro and macro levels, one cannot expect similar spatial patterns over time. However to say that there is a resurgence is not clear. Ideally one would expect spatial heterogeneity when there are areas with low levels of infection with few having high levels. Lines:
17. Lines: 378-381: The authors say “Spatial distribution may continue to change as prevalence changes, e.g. reduction in prevalence after MDA, or increasing prevalence if persistent resurgence” – any reference to support the latter part of the phrase regarding resurgence ? please provide
18. Lines: 384-391: One ought to see the sample sizes of the area Fagalil where only 38 were tested (Lau et al. 2017) and is termed as hotspot. In that study, the design was convenience sampling. So it really makes wonder whether the samples were drawn from an area were infections were known to be present. The last sentence is contradicting because they have mentioned that the infection levels were lower than the recommended threshold levels and it has now resurged. And here suddenly a mention of “reintroduction of parasites” . The statements are confusing
19. Lines: 402-04: The statement is not clear. Further, being positive for Ab need not necessarily mean that they should have either Mf or the filarial adult worm. Also, it depends on which Ab tool is being used. While Wb123 Ab measures the exposure to incoming L3 larvae, Bm14 is specific for presence of Adult worm (with or without Mf). However, Bm33 is not very specific as it is cross reactive to other parasites as well. In such a situation, this statement does not hold all times.
20. Lines: 421-22: Does this statement indicate that Bm14 and Bm33 measure old infections only? Clarify
21. Lines: 428-430: Vague statement. “True clusters” – what does this mean
22. Lines: 433- 444: After doing all the analysis, the authors final say what each spatial analytical method can and cannot do. Ideally, this should be mentioned in the methodology itself. After having given so much credit for SaTScan in the previous paragraphs, the sentence in 438-39 cautions the interpretations.
23. Lines: 446-447: Ag need not be only active or recent infection. Remnants of dead adult worms their secretions could also be measure by Ag. In addition, the authors themselves have accepted regarding what the Ab tests detect. In such a situation, one cannot assume it would also mean Ag and or Mf positivity.
24. Lines: 451-453: Are we interested in knowing clusters that were formerly active? Why? The need of the hour is to identify those emerging or those with residual transmission. The authors may highlight what additionally this analysis has helped to achieve.
25. Lines: 463-466: The authors have used 3 approaches of spatial analysis: semi-variograms, Getis Ord G statistics and SatScan to demonstrate spatial dependency, hotspots and high risk clusters. Ultimately say each indicate different information.
26. Lines: 467-472: The authors say “The spatial heterogeneity …………….highly localised and spatially-explicit factors, e.g. climatic and ecological factors, human behaviour, mosquito distribution and density, previous MDA coverage, or a combination of these factors. The American Samoa is a small group of islands – would one expect any drastic change in climatic and ecological factors?
27. The authors say these methods to be embedded for identifying transmission areas but in most discussion part, these methods have only identified those hotspots that were already identified by earlier surveys. IN addition to the already mentioned hotspots, what are the new areas that were detected by these methods? A table showing how many sites were identified as hotspots or part of risk clusters could be shown.
28. Line 477-82: What was the utility of the non-spatial methods in assessing clustering and how this information could be used…this may be discussed as well

**Additional comment**

- Additionally in the current situation, it is not only necessary to see only the high risk clusters but also the low prevalences areas as identified by Getis Ord G. The range of prevalences of Mf, Ag, Ab in these high-high and low-low clusters could be shown in the a table. It would be interesting to see if these methods that identified low-low clusters for all indicators. If so are these below the recommended thresholds (say 1% for MF and 2% for Ag, however for Ab we do not have any thresholds so far). Adding Moran’s L along with these would add value to this manuscript.
- The manuscript should indicate what additional information has been provided by this analysis and how this would be useful in terms of achieving LF elimination goal.
