# Supplementary material for: Lymphatic filariasis in 2016 in American Samoa: Identifying clustering and hotspots using non-spatial and three spatial analytical methods
Source: PLoS Negl Trop Dis. 2022 Mar 28;16(3):e0010262. doi: 10.1371/journal.pntd.0010262 (PMC8989349; doi:10.1371/journal.pntd.0010262)
Supplement: Attachment — Submitted filename: Reviwers_Comments_final.docx [file pntd.0010262.s005.docx]

19 October 2021

Editor-in-chief

PLoS NTD

Ref: Submission of revised manuscript no PNTD-D-21-00923.

To Editor-in-Chief,

I, on behalf of my co-authors, would like to submit the revised manuscript “Lymphatic filariasis in 2016 in American Samoa: Identifying clustering and hotspots using non-spatial and three spatial analytical methods”.

All the reviewer’s comments have been addressed and responses have been outlined in the attached Responses to Reviewer's document.

Thank you for your consideration.

Sincerely,

(Dr Kinley Wangdi)

Corresponding author

**Reviewer #1**

**General comments:** This is a well written manuscript that illustrates how spatial and non-spatial analysis can be used to assess clustering of biomarkers, identify hotspots, and compares the various methods. I found the authors' step by step description of the different analyses in the methods section very helpful and appreciated the level of detail included.

**Response:**

Thank you for the positive comments

1. **Methods**
   -Are the objectives of the study clearly articulated with a clear testable hypothesis stated?
   -Is the study design appropriate to address the stated objectives?
   -Is the population clearly described and appropriate for the hypothesis being tested?
   -Is the sample size sufficient to ensure adequate power to address the hypothesis being tested?
   -Were correct statistical analysis used to support conclusions?
   -Are there concerns about ethical or regulatory requirements being met?

**Reviewer #1:** The authors present the spatial and non-spatial methods in a clear manner for readers who may not be familiar with such analyses. The dataset is rich with household GPS coordinates, and antigen, microfilariae, and antibody data for ~2500 participants from 30 villages in American Samoa.
**Response:** Thank you.

2. **Results**
   -Does the analysis presented match the analysis plan?
   -Are the results clearly and completely presented?
   -Are the figures (Tables, Images) of sufficient quality for clarity?
   **Reviewer #1:** The results presented in this manuscript are clear and follow the analysis plan described in the methods. The figures and tables match the data presented in the results section of the paper and are presented quite nicely.
   **Response:** Thank you for your comments.

-There is one figure, Figure 6, which is out of order at the end of the manuscript where all figures are presented.
**Response**: Figure 6 has been correctly uploaded in the revised manuscript.

3. **Conclusions**
   -Are the conclusions supported by the data presented?
   -Are the limitations of analysis clearly described?
   -Do the authors discuss how these data can be helpful to advance our understanding of the topic under study?
   -Is public health relevance addressed?

**Reviewer #1:** The authors' interpretations of the results are thorough and the conclusions are supported by the data presented. An added strength is the comparison to the known hotspots from the previous studies. The authors clearly present the limitations of the study and how the analytical methods can be used.

In the authors summary, the authors note that 'embedding the tools used in this study into operational planning for LF surveillance may identify ongoing transmission not identified through routine surveys'. I think it would be helpful to describe a bit about the feasibility of this (i.e. do country programs generally have the capacity to do such analyses). Also, I would be curious to know how the American Samoa LF elimination program plans to use these data if there is a plan to do so.

**Response:** Thanks for highlighting this. Some countries would not have the capacity to undertake such analysis, but many countries are starting to use Geographic Information Systems (GIS) including spatial decision support systems for operational activities. In recent years, many user-friendly software (including freeware) platforms have been developed to enable countries to collect spatially-explicit data during surveys, and to act on results in a timely manner. Examples include Standard Data (previously Secure Data Kit, <https://www.standardco.de>) and Kobo Toolbox (<https://www.kobotoolbox.org>). The American Samoa program has used information on village level prevalence and known hotspots in survey design since 2016. Currently, there are no official guidelines on how best to detect or respond to hotspots. Our study is a first step to generating evidence on possible methods that can be used, and the potential value of the findings for elimination programs.

4. Line 72 states 120mil people are infected with LF, but a recent WER updated this number to 51.4 million as of 2018. LF- WER9543 Global programme to eliminate lymphatic filariasis: progress report, 2019.

**Response:** We have updated the manuscript with the latest figures:

**Page 4, line numbers 73-74:** Lymphatic filariasis (LF) is a neglected tropical disease, with an estimated 51.4 million people infected in tropical and subtropical areas globally in 2018 [1].

**Reviewer #2**

**General comments:** Need improvement. Write up is a bit confusing at certain places.

1. **Methods**
   -Are the objectives of the study clearly articulated with a clear testable hypothesis stated?
   -Is the study design appropriate to address the stated objectives?
   -Is the population clearly described and appropriate for the hypothesis being tested?
   -Is the sample size sufficient to ensure adequate power to address the hypothesis being tested?
   -Were correct statistical analysis used to support conclusions?
   -Are there concerns about ethical or regulatory requirements being met?
   **Reviewer #2:** Some additional analysis is required to add value to the manuscript

**Response:** Responses to specific questions have been provided below.

2. **Results**
   -Does the analysis presented match the analysis plan?
   -Are the results clearly and completely presented?
   -Are the figures (Tables, Images) of sufficient quality for clarity?

**Reviewer #2:** Additional tables requested

**Response:** We have added a table with results of Moran’s I statistics (Supplementary Table 1).

3. **Conclusions**
   -Are the conclusions supported by the data presented?
   -Are the limitations of analysis clearly described?
   -Do the authors discuss how these data can be helpful to advance our understanding of the topic under study?
   -Is public health relevance addressed?

**Reviewer #2:** Needs to be strengthened
**Response:** We have provided additional details in the responses below, and in the revised manuscript.

4. The authors have used 5 infection indicators Mf, Ag, Wb123, Bm14 and Bm33. It would be useful for the reader if they also indicate what each indicator measures, as this has implications later while discussing about their spatial patterns.
a) How useful is B33 as this is known for its cross reactivity with other parasites?

**Response:** We have now included this information in methods section.

**Page 8, line numbers 172-180:** Mf positivity represents active infection and infectiousness. Ag indicates the presence of live or dead adult filarial worms in the lymphatic system, and may persist for months or years after treatment [31, 32]. The Wb123 Ab was identified by a library generated from *W. bancrofti* L3 larval stages [33]; it appears in the early stages of infection and persists for long periods after infection; antibody dynamics post-infection and post-treatment are not well understood, and may differ between adults and children [34]. The Bm14 Ab was identified from a cDNA library screened using sera from microfilariaemic people [35, 36]. Bm33 Ab was also identified in a *B. malayi* cDNA library as a major cross-reacting immunogen in *W. bancrofti* [37]. Bm14 and Bm33 Abs may persist long (many years) after infection, but exact duration is not well known.

b) A table with site wise details of samples size, Mf, Ag and the different Ab prevalence could be provided so that it would be useful while interpreting the findings of the spatial analysis

**Response:** In the current paper, the spatial analyses considered American Samoa as one continuous spatial surface (not stratified by village), so village level data are not critical for interpretation of results. For readers who are interested in village level data, these are available from our previous publication (Sheel et al., Identifying residual transmission of lymphatic filariasis after mass drug administration: Comparing school-based versus community-based surveillance - American Samoa, 2016. PLoS Negl Trop Dis. 2018; 12:e0006583).

5. Line 124-25: The authors say the adjusted Ag prevalence- what do they mean by that? How was that estimated after adjusting for which variables – the authors may explain briefly in few lines.

**Response:** We have added text as below:

**Page 6, line numbers 125-128:** In this paper, we use the results of the 2016 community-based survey in American Samoa to investigate the spatial epidemiology of LF when there was strong evidence of resurgence (after adjusting for survey design, age and sex, the estimated Ag prevalence in 2016 was 6.2% (95% CI 4.5-8.6%) in residents aged >8 years [18, 19]).

6. Lines 108-113: This is nothing new, as it is expected that the older age classes will have higher infection levels compared to 6-7 yo, due to their prolonged exposure (even prior to the MDAs). But how does one interpret whether these are really due to on-going transmission as the Ag and Ab status do remain positive even after repeated rounds of MDA, even when Mf is zero or levels are very low.

**Response:** It is true that older persons will likely have higher antigen prevalence due to greater exposure time. However, as we have data from previous years (2010 and 2014) where we found lower antigen prevalence, we were able to demonstrate an increase in Ag prevalence over time. The detection of Mf in some participants also confirmed ongoing transmission. Furthermore, American Samoa failed school-based TAS-3 in 2016 (after passing TAS-1 and TAS-2 in previous years), providing another indication of ongoing transmission and resurgence.

7. Lines 234-246: The authors may kindly include a statement that approval was obtained to take blood samples (mention quantity) from the participants in the study.

**Response:** The ethical approval included the permission to take blood samples, as noted in our previous paper (Sheel et al 2018).

**Page 12, line numbers 268-270:** A signed informed consent to collect demographic data and blood samples was obtained from adult participants or from parents/guardians of the participants <18 years, along with verbal assent from minors [21].

8. Lines 274-277: Did the authors try fitting any distributions to the frequency distribution of no. of infected persons per household for different indicators? (Like Poisson or Negative binomial). It can be tried as well as this also will give an idea on the distribution pattern of cases for different indicators at household and also at site level.

**Response:** This is an interesting suggestion for future examination, but within-household distribution of positives is not the focus of this work. We did not fit the distributions of the frequency of number infected persons per households for different indicators, since our focus is on clustering between households/sites. We don’t believe that this analysis will be useful for this paper because all participants within each household have the same spatial location. Nevertheless, we have provided the distributions of households or survey locations with 0, 1, 2 and >2 infected persons (by indicator) in Supplementary Tables 2 and 3 for additional information.

9. Lines: 284-288: As the authors have used mixed effects LR model with households and villages as random effects in their respective models, the Table 1 to include the significance of the ICC estimated for different infection indicators.
a. What did the authors interpret from the clustering within households or villages? What could be the reasons for such clustering?

**Response:** Our results showed that clustering was stronger at household than village level for all infection markers. The results suggest that transmission between household members is more likely than transmission between residents of the same village. This finding is consistent with previous observations of highly localised hotspots. The finding is also supported by the fact that the main vector for LF in in America Samoa, *Aedes polynesiensis*, has a short average flight range of around 100 meters and breeds easily in household reservoirs.

**Page 16, line numbers 383-386:** This is plausible because *Aedes polynesiensis*, the main vector for LF in America Samoa, has a short flight range of ~100 meters [42]. These results corroborate our findings in neighbouring Samoa, where ICC for Ag and Mf were higher for households (0.46 and 0.63) compared to PSUs (0.18 and 0.12) [43].

b. A multilevel analysis accounting for village and household will help in assessing the contribution of variation due to different levels – could be carried out so that appropriate interventions could be undertaken at different levels.

**Response:** Results of multilevel logistic regression were presented in a previous paper (Lau et al. 2020. https://journals.plos.org/plosntds/article?id=10.1371/journal.pntd.0008916). In the current study, we focussed on non-spatial and spatial indicators of clustering of five infection markers. We have also provided ICC for each infection marker at household and village levels (Table 1) – these results were generated from a mixed effects (multilevel) logistic regression model.

10. Lines-290-295: The authors give semivariogarms and its parameters of spatial autocorrelation like sill, partial sill, nugget etc. Instead, it is ideal to estimate the spatial autocorrelation (Moran’s I) and also assess its significance before interpreting the parameters like sill, partial sill etc and giving the size of the clusters. This has to be estimated for each of the infection indicators to see if the spatial dependency is really significant.

**Response:** We agree with the reviewer and Moran’s I have been added as supplementary Table 1. Moran’s I show that spatial autocorrelation for all the infection markers were significant. Semivariograms give results for cluster size, which is not provided by Moran’s I statistics.

**Page 10, line numbers 220-221:** The global significance of clusters of infection markers were also assessed using Moran’s I Statistics (**Supplementary Table 1**).

11. Fig 7 - The semivariograms for Ag and Mf do not show any pattern of spatial dependency compared to the Ab, though it is mentioned there is clustering. A Moran’s I would be more useful here to assess.

**Response:** We agree with the reviewer and Moran’s I statistics have been added as Supplementary Table 1. Moran’s I show that there was significant spatial autocorrelation for all the infection markers. Semivariograms give results for cluster size, which is not provided by Moran’s I statistics.

12. Lines: 296-305:
a. How was the window size decided for SaTScan tool?

**Response:** The window size in SatScan was set to include a maximum of 25% of the observations refer to methods. This information has been added to Methods.

**Page 10, line numbers 236-238:** SaTScan was set to include a maximum of 25% of the observations within circular windows, and a Bernoulli model was used because the outcome variables of interest were binomial (positive and negative results).

b. From the results its understood all analysis were at household level ie household geolocations?

**Response:** That is correct, the spatial analysis was done at the household level. There were 750 households in 730 unique locations because some households shared the same structure (e.g. apartments). This has been clarified.

**Page 12, line numbers 281-282:** The study included 750 households at 730 unique survey locations; some households shared the same structure, e.g. apartment buildings.

c. Since there were 30 sites, was clustering identified at village level also? If so what were the results.

**Response:** There were 32 primary sampling units (30 villages) that included 750 households. We did not conduct spatial analysis at the village level because the survey was designed to select a population proportionate sample from each primary sampling unit, and very few households were selected in the smallest villages. Spatial analyses were done by considering the whole island as one surface, and using household locations as data points. When describing results, we referred to villages that fell within the significant clusters.

d. When more than one cluster is formed, which cluster is more significant and what differs between different clusters, in terms of range of prevalence.

**Response:** The clusters were ranked based on the log likelihood ratio (LLR), and clusters with higher LLR were associated with higher relative risk. The following clarification has been added.

**Page 11, line numbers 245-246:** The clusters were ranked based on the log likelihood ratio (LLR), and those with higher LLR were associated with higher relative risk.

e. What was the range of observed prevalence of different indicators in the clusters identified by SaTScan? It would be interesting to see how these clusters are. A Table would suffice.

**Response:** The prevalence of different indicators in the clusters from SaTScan are presented in **Table 3**.

13. Lines 306-316: If correct, Getis-Ord General G tool is most appropriate there is an even distribution of values with spatial spikes of high values. However, when both high and low values cluster in space, measuring spatial clustering using spatial autocorrelation is a better tool which also has means to test if those clusters are significant as well. Since all the infection indicators are showing both low and high clusters, more appropriate tool to study spatial heterogeneity would be estimate the spatial autocorrelation statistics and interpret the patterns based on their significance.

**Response:** We believe that the Getis-Ord Gi* statistics is better suited for our dataset because both high and low values cluster spatially. Please refer to: <https://help.arcgis.com/en/arcgisdesktop/10.0/help/#/How_Hot_Spot_Analysis_Getis_Ord_Gi_works/005p00000011000000/>

<https://help.arcgis.com/en/arcgisdesktop/10.0/help/#/How_Cluster_and_Outlier_Analysis_Anselin_Local_Moran_s_I_works/005p00000012000000/>

14. Lines 377-381: Authors say in 2016 it’s a resurgence –
a. How does one define resurgence?
b. What are the associated cut-off levels for these different indicators?
c. Authors to kindly explain these with appropriate references.
d. This contradicts with the statement in lines: 59-60 where they address these as residual hotspots and areas with on-going transmission. The authors should be very clear in what they want to emphasize.

**Response:** This paragraph has been revised as per the suggestion from another reviewer.

a) Resurgence is a commonly used term to indicate significant increase in infection prevalence to levels above target thresholds, indicating increase in transmission. (e.g. Prada et al. https://academic.oup.com/jid/article/221/Supplement_5/S503/5680710?login=true)
b) Cut-offs for the infection indicators were based on levels determined by the CDC, and described in previous publications (e.g., Won et al. <https://journals.plos.org/plosntds/article?id=10.1371/journal.pntd.0006347>)
c) The above references have been added to the manuscript.
d) ‘Residual hotspots’ and ‘areas of ongoing transmission’ are not mutually exclusive. Ongoing transmission occurs at these residual hotspots.
15. Lines: 343-45: The authors should explain why the results varied with valid interpretations in terms of indicators and the methods used by different spatial analytical methods.

**Response:** We have summarised the main findings in this paragraph and the possible explanations for these findings have been provided in the subsequent paragraphs.

16. Lines: 346-47: the authors say “identified significant spatial dependency for all infection markers, with different cluster size for each marker”. Was any significance testing done? Those details are not given in the results. Please provide them

**Response:** The levels of significance of spatial dependency for different infection markers using the four methods are presented in results section and **Tables 2 and 3**. We have made it clearer that all semivariogram results presented in Table 2 were statistically significant. Table 3 includes p values for each significant cluster identified by SaTScan.

**Page 14, line numbers 323-333:** Using Kuldorff’s scan statistic (SaTScan), significant clustering of all infection markers was identified around Fagali’i village (cluster 1) in the north west, an area of high Ag prevalence identified by our previous studies from 2010 and 2014 (**Fig 2b, 3b, 4b, 5b, 6b)**. RR was highest for Mf (41.46, p<0.0001), followed by Ag (16.10, p<0.0001), Bm14 Ab (6.13, p<0.0001), Wb123 Ab (3.43, p<0.0001), and Bm33 Ab (2.13, p<0.0001) (**Table 3).** The Ag cluster had the smallest radius (0.44 km) while clusters were larger for Mf (1.85 km) and all three Abs (2.31 km) (**Table 3**). Significant clustering of Wb123, Bm14 and Bm33 Abs (RR=2.68, p<0.001; 3.15, p=0.0025 and 1.41, p<0.001) were identified in Vaitogi (cluster 2), a previously known area of high Ag prevalence (**Fig 4b, 5b, 6b)**. Clustering of Bm33 Ab was also identified in Ili’ili-Tafuna (cluster 3) adjacent to Vaitogi (RR=1.64, p=0.015) and in the Pago Pago-Anua area (cluster 4, RR=1.69, p=0.044, **Fig 6b).**

17. Lines: 353-358: A multilevel analysis could help in assessing which level is contributing more to the variation in these infection indicators.

**Response:** We conducted mixed effects (multilevel) logistic regression to determine the ICCs at village and household levels. The results in Table 1 show that clustering is more significant at household than village level for all infection markers. Multilevel analysis of other variables were presented in an earlier publication (Lau et al, 2020. https://journals.plos.org/plosntds/article?id=10.1371/journal.pntd.0008916). In this paper, we focused on clustering of infection markers using both non-spatial (ICC) and spatial measures.

18. Lines: 358-360: Is identifying clusters of positive individuals only for testing and treating them? How about using these information for targeted intervention?

**Response:** The reviewer raises a very good question. Currently, there are no official recommendations on how best to manage clusters and hotspots. We agree that information about clusters could potentially be used for targeting interventions. However, the ability to identify clusters is a challenge in itself, and in this paper, we explored multiple methods for doing this. Understanding the spatial epidemiology of clusters in the post-elimination setting would also be helpful for developing surveillance strategies, e.g. spatial sampling designs.

**Page 16, line numbers 387-389:** Strong clustering of infected persons within households suggests that household members of Ag-positive and Mf-positive persons should be offered testing and/or treatment as part of surveillance activities [44, 45].

19. Lines: 366-371: With the dynamics of environment both at micro and macro levels, one cannot expect similar spatial patterns over time. However, to say that there is a resurgence is not clear. Ideally one would expect spatial heterogeneity when there are areas with low levels of infection with few having high levels.

**Response:** Resurgence was not determined based on changes in spatial pattern over time. Prior to this study, resurgence of LF in American Samoa had already been confirmed through

multiple sources of evidence (please see above point 11). In this study, we applied multiple methods to determine the spatial patterns of infection markers at difference stages of transmission.

20. Lines: 378-381: The authors say “Spatial distribution may continue to change as prevalence changes, e.g. reduction in prevalence after MDA, or increasing prevalence if persistent resurgence” – any reference to support the latter part of the phrase regarding resurgence? please provide.

**Response:** This is a comment in the discussion, and reflects what the authors believe may happen as prevalence changes and respreads from hotspots into additional or formerly higher prevalence areas. The changing pattern of hotspots between 2010 and 2016 is suggestive of this, and it is also one possible explanation for changes in frequency of haplotypes observed between 2014 and 2016 (Hedtke et al 2020).

21. Lines: 384-391: One ought to see the sample sizes of the area Fagalil where only 38 were tested (Lau et al. 2017) and is termed as hotspot. In that study, the design was convenience sampling. So it really makes wonder whether the samples were drawn from an area where infections were known to be present. The last sentence is contradicting because they have mentioned that the infection levels were lower than the recommended threshold levels and it has now resurged. And here suddenly a mention of “reintroduction of parasites”. The statements are confusing.

**Response:** Fagali’i is a very small village with population of ~250 people. Even though only 38 were tested in 2014, this represents 15% of the total population (information on village population was provided in Lau et al 2017). The last sentences referred to by the reviewer reads “Identification of an Ag cluster in this area from 2010 data (and confirmed in 2014) suggests that transmission may have been occurring here for many years. However, it is not possible to determine whether transmission had never been interrupted by MDA in the early-mid 2000s, or if it had ceased at some point but restarted due to the reintroduction of parasites”. We have reworded this to read:

**Pages 17-18, line numbers 412-419:** Fagali’i was first identified as a potential hotspot through a serological study of samples collected in 2010 [19] for a population proportionate survey of American Samoa. Repeat surveys in 2014 and 2016 confirmed very high Ag prevalence [18, 21]. MDA was conducted in American Samoa from 2000 to 2006. Based on the results of these cross-sectional studies (2010, 2014, 2016), it was not possible to determine if transmission in Fagali’i had been interrupted by the rounds of MDA in the early-mid 2000s but restarted later due to reintroduction of parasites to the village, or whether transmission was never interrupted by MDA.

22. Lines: 402-04: The statement is not clear. Further, being positive for Ab need not necessarily mean that they should have either Mf or the filarial adult worm. Also, it depends on which Ab tool is being used. While Wb123 Ab measures the exposure to incoming L3 larvae, Bm14 is specific for presence of Adult worm (with or without Mf). However, Bm33 is not very specific as it is cross reactive to other parasites as well. In such a situation, this statement does not hold all times.

**Response:** In this sentence, we were referring to the epidemiological associations rather than the biological explanations. We do not accept necessarily that Wb123 antibody only reflects exposure to L3, or that Bm14 is specific to adult worms. We have reworded the sentence to read: “These examples shows that from an epidemiological perspective, clustering of Abs could be useful for identifying Ag-positive and Mf-positive persons”.

**Page 18, line numbers 432-434:** These examples shows that from an epidemiological perspective, clustering of Abs could be useful for identifying Ag-positive and Mf-positive persons.

23. Lines: 421-22: Does this statement indicate that Bm14 and Bm33 measure old infections only? Clarify

**Response:** We have revised the manuscript to include the reviewer’s concerns.

**Page 8, line numbers 172-180:** Mf positivity represents active infection and infectiousness. Ag indicates the presence of live or dead adult filarial worms in the lymphatic system, and may persist for months or years after treatment [31, 32]. The Wb123 Ab was identified by a library generated from *W. bancrofti* L3 larval stages [33]; it appears in the early stages of infection and persists for long periods after infection; antibody dynamics post-infection and post-treatment are not well understood, and may differ between adults and children [34]. The Bm14 Ab was identified from a cDNA library screened using sera from microfilariaemic people [35, 36]. Bm33 Ab was also identified in a B. malayi cDNA library as a major cross-reacting immunogen in *W. bancrofti* [37]. Bm14 and Bm33 Abs may persist long (many years) after infection, but exact duration is not well known.

**Page 19, line numbers 446-448:** The absence of Bm14 and Bm33 Ab hotspots in this area may indicate that more recent transmission than clusters 2 and 3 because these antibodies persist for years.

24. Lines: 428-430: Vague statement. “True clusters” – what does this mean

**Response:** We have removed the term “true clusters”, and reworded as follows:

**Page 19, line numbers 453-455:** For the SaTScan results, high prevalence of all infection markers (including Ag and Mf) within most clusters (**Fig 8**) suggest that they represent areas of ongoing transmission.

25. Lines: 433- 444: After doing all the analysis, the authors final say what each spatial analytical method can and cannot do. Ideally, this should be mentioned in the methodology itself. After having given so much credit for SaTScan in the previous paragraphs, the sentence in 438-39 cautions the interpretations.

**Response:** The different methods serve different purposes. The statistics underlying different methods have been added under the Methods.

**Page 10, line numbers 225-228**: The Kulldorff’s scan statistics were used to determine the geographic distribution of clusters of high prevalence of infection markers, while Getis-Ord Gi* statistic was used to test the statistical significance of hotspots and to determine the spatial dependence and relative magnitude between observations.

26. Lines: 446-447: Ag need not be only active or recent infection. Remnants of dead adult worms their secretions could also be measure by Ag. In addition, the authors themselves have accepted regarding what the Ab tests detect. In such a situation, one cannot assume it would also mean Ag and or Mf positivity.

**Response:** We agree with the reviewer that Ag positivity is non-specific in regards to the status of infection, but is generally detected when there is current or recent presence of adult worms (including remnants of dead worms). For this reason, Ag is routinely used as the standard indicator in LF programmatic surveys. We have not indicated anywhere in the manuscript that Ab positivity means Ag or Mf positivity. We have highlighted the limitations of Ag and Abs in the manuscript.

27. Lines: 451-453: Are we interested in knowing clusters that were formerly active? Why? The need of the hour is to identify those emerging or those with residual transmission. The authors may highlight what additionally this analysis has helped to achieve.

**Response:** We agree with the reviewer that the main focus should be to find current hotspots. Our analyses show that antibodies are potentially useful for identifying clusters of Ag-positive and Mf-positive people, and hence useful for identifying currently active clusters. The spatial epidemiology of former clusters is also useful for understanding the transmission dynamics of LF. As mentioned above, one of the current programmatic challenges is the ability to find such clusters; our study is a preliminary investigation of the different analytical methods that could be applied, as well as the value of using a combination of infection markers rather than relying only on Ag. As seen with our example of American Samoa, resurgence could potentially have been detected earlier if Abs were used together with Ag.

28. Lines: 463-466: The authors have used 3 approaches of spatial analysis: semi-variograms, Getis Ord G statistics and SatScan to demonstrate spatial dependency, hotspots and high risk clusters. Ultimately say each indicate different information.

**Response:** This study looked at three different methods to study spatial heterogeneity and compared and contrasted these methods. The conclusion from all the three methods can be used for spatial analysis and the choice depends on the type of questions.

29. Lines: 467-472: The authors say “The spatial heterogeneity …………….highly localised and spatially-explicit factors, e.g. climatic and ecological factors, human behaviour, mosquito distribution and density, previous MDA coverage, or a combination of these factors. The American Samoa is a small group of islands – would one expect any drastic change in climatic and ecological factors?

**Response:** Even though American Samoa is very small, climate and environment differ between east and west, and between coastal versus mountainous areas (highest altitude ~650m on Tutuila). In a previous study of leptospirosis in American Samoa, environmental factors (land use, vegetation, etc) were significant predictors of leptospirosis hotspots, highlighting that small-scale spatial variation in these factors are important drivers of the transmission of infectious diseases. (https://journals.plos.org/plosntds/article?id=10.1371/journal.pntd.0001669).

30. The authors say these methods to be embedded for identifying transmission areas but in most discussion part, these methods have only identified those hotspots that were already identified by earlier surveys. In addition to the already mentioned hotspots, what are the new areas that were detected by these methods? A table showing how many sites were identified as hotspots or part of risk clusters could be shown.

**Response:** This paper is an exploration of new approaches to identifying clusters and hotspots, using results of our field studies to verify the accuracy of these approaches. For example, would we have identified resurgence earlier if we used antibodies as well as Ag and Mf? The study also highlights the value of household surveys, which are not routinely done in post-MDA surveillance, for identifying hotspots. This study confirmed known hotspots (line numbers 421-423) as well as identified new hotspots (line numbers 436-438). A summary of clusters and hotspots is provided in **Tables 2** and **3**.

**Page 18, line numbers 420-422:** Both local spatial methods also identified clusters or hotspots in a previously known hotspot around Ili’ili-Vaitogi-Futiga (cluster 2). Getis-Ord Gi* identified hotspots of all infection markers in this area, while SaTScan identified significant clusters of the three Abs.

**Page 18, line numbers 435--437:** SaTScan and Getis-Ord Gi* identified two other clusters of Bm33 Ab (**Fig 2c, 6b and 6c**). One in Ili’ili-Tafuna (cluster 3), east of our previously identified hotspot in Ili’Ili/Vaitogi/Futiga. The other is a newly identified cluster in the Pago Pago-Anua area (cluster 4).

31. Line 477-82: What was the utility of the non-spatial methods in assessing clustering and how this information could be used…this may be discussed as well.

**Response:** The use of non-spatial methods is covered in the Methods (Page 8, lines numbers 185-190). Non-spatial method (ICC) provides a general measure of the level of clustering at household and village levels across the whole study area, while spatial methods show location specific information.

**Page 8, line numbers 185-190:** The ICC provides a measure of how strongly similar observations are clustered across the households and villages. As the variable of interest (e.g. Ag-positive) becomes more homogeneous at the village or household levels (i.e. stronger clustering), the ICC tends to approach one. Conversely, as variables become more heterogeneous, the ICC approaches zero. ICC does not take into account the spatial distribution or geographic locations of households or villages.

**Additional comments**

32. Additionally, in the current situation, it is not only necessary to see only the high risk clusters but also the low prevalences areas as identified by Getis Ord G. The range of prevalences of Mf, Ag, Ab in these high-high and low-low clusters could be shown in a table. It would be interesting to see if these methods that identified low-low clusters for all indicators. If so are these below the recommended thresholds (say 1% for MF and 2% for Ag, however for Ab we do not have any thresholds so far). Adding Moran’s I along with these would add value to this manuscript.

**Response:** We have added results of Moran’s I, and there was significant spatial autocorrelation for all infection markers. We could add coldspots for SaTScan but these results are unlikely to be of any value to programme activities because target thresholds are currently defined for whole populations rather than small geographic areas such as those identified as cold spots. Also because of the high level of movement of people within and between the islands (Xu et al. 2018: https://www.nature.com/articles/s41598-018-28081-x) and potential spread from hotspots, the programme cannot exclude them from surveys.

33. The manuscript should indicate what additional information has been provided by this analysis and how this would be useful in terms of achieving LF elimination goal.

**Response:** Our manuscript provided multiple advances in knowledge that are relevant to LF elimination:

- We explored the use of spatial and non-spatial methods for measuring clustering at household and village levels, and for identifying locations of clusters. Non-spatial method (ICC) provides a general measure of the level of clustering at household and village levels across the whole study area, while spatial methods show location specific information.
- We confirmed that these methods identified known clusters, as well as new clusters
- We have provided evidence for the potential utility of Abs in LF surveillance, including the use of Ab clusters to identify Ag and Mf clusters (line numbers 436-438).

**Reviewer #3: Minor comments**

**General comments:** This ms aims to compare the usefulness of different spatial (Kuldorf’ scan statistic, Getis-Ord G* & variogram) and non-spatial (ICC) methods to characterize the clustering and hotspots of LF in American Samoa, which passed school-based TAS 1 and 2 after 7 rounds of MDA. Subsequently in 2016, a separate operational research study which compared the results of school-based TAS-3 and a community-based survey indicated resurgence of LF after passing TAS 2. In the present study, the authors using the data from 2016 community-based survey, demonstrated the usefulness of the spatial and non-spatial analytical methods for identifying LF clusters and hotspots in terms of Ag, and three antibody markers (Wb123, Bm14 &Bm33) in American Samoa. The ms is well written, with clear objectives, applied appropriate statistical methods and the results are presented with appropriate tables and figures. The Discussion is too lengthy to read, it is desirable to shorten it. Altogether, I would recommend the ms for publication after a revision based on the following minor comments.

Response: Thanks for these comments. The Discussion has been shortened by removal of repetitive information in several paragraphs and by cutting unnecessary words. However, the nature of the study, which used one non-spatial and three spatial methods, five infection markers, and identified at least 4 clusters with different combinations of markers means that the Discussion has to be relatively lengthy to cover all this material.

1. **Methods**
   -Are the objectives of the study clearly articulated with a clear testable hypothesis stated?
   -Is the study design appropriate to address the stated objectives?
   -Is the population clearly described and appropriate for the hypothesis being tested?
   -Is the sample size sufficient to ensure adequate power to address the hypothesis being tested?
   -Were correct statistical analysis used to support conclusions?
   -Are there concerns about ethical or regulatory requirements being met

**Reviewer #3:** Yes to all.

**Response:** Thank you.

2. **Results**
   -Does the analysis presented match the analysis plan?
   -Are the results clearly and completely presented?
   -Are the figures (Tables, Images) of sufficient quality for clarity?
   **Reviewer #3:** Yes study results match with the statistical analysis plan. Specific comments are given in the attached Reviewer's comments.

**Response:** Thank you.

3. **Conclusions**
   -Are the conclusions supported by the data presented?
   -Are the limitations of analysis clearly described?
   -Do the authors discuss how these data can be helpful to advance our understanding of the topic under study?
   -Is public health relevance addressed?

**Reviewer #3:** Yes to all. The conclusions are supported with data. Study limitations are clearly given and discussed how further methodological improvements would be helpful to have a better understanding the topic of the study.
**Response:** Thank you.

4. Line 39: insert “spatial” before “clustering”.

**Response:** We have added “spatial” before clustering in the revised manuscript.

**Page 2, line numbers 40-42:** Using Kuldorff’s scan statistic, significant spatial clustering was observed in two previously known locations of ongoing transmission: for all markers in Fagali’i and all Abs in Vaitogi.

5. Line 98: please mention the drug regimen used in the MDA rounds.

**Response:** We added the drug regimen as follows.

**Page 5, line numbers 98-101:** American Samoa has made efforts to eliminate LF through two MDA programs. Firstly, in 1963 and 1965 [13] with repeated doses of diethylcarbamazine (DEC), and secondly as part of PacELF [14], seven MDA rounds were distributed between 2000 and 2006 using single annual doses of DEC plus albendazole [14].

6. L137-139: The description given in this para gives the impression that the data used in the present study were collected based on both the WHO recommended TAS and multistage cluster survey as described by Sheel et al. Please make it clear that the study by Sheel et al (2018) collected data in 2016 via (i) WHO recommended systematic school-based TAS and (ii) two-stage (rather than multi-stage, can be specific to say as two-stage: stage 1: cluster (village) and stage 2: household) community-based household survey. The authors have to clearly state that the present study used the data from community-based household survey.

**Response:** Thanks for this correction and we agree that current WHO recommendations cover only TAS. We have updated the manuscript to make it clear by adding the suggested changes.

**Page 6, line numbers 140-141:** Briefly, the study used a two stage community-based probability cluster survey, where clusters (primary sampling units [PSUs]) were selected in stage one and households in stage two.

7. Lines 176-182: The para may be shortened by explaining the method and its usefulness for spatial dependency. The sentences between lines 176-182 may be deleted as they give more of description about semivariogram. The sentence starting from “Outputs from …..location of hotspots” may be moved to the end of next para after line 199.

**Response:** The suggested edits have been made in the revised manuscript.

8. L184-185: The authors can also calculate the Local Moran’s I, a local indicator of spatial association (LISA), to identify the local spatial clusters. It measures the degree of spatial autocorrelation at each specific location (Anselin, 1995: Local indicators of spatial association – LISA. Geogr. Anal., 27, 93–115) as it compares only the neighboring value rather than the overall mean. A high positive local Moran’s I value implies the target value is similar to its neighborhood, and then the locations are spatial clusters, which include high–high clusters (high values in a high value neighborhood) and low–low clusters (low values in a low value neighborhood). Meanwhile, a high negative local Moran’s I value implies a potential spatial outlier, which is obviously different from the values of its surrounding locations.

**Response:** We have included results of Moran’s I in **Supplementary Table 1**, showing significant autocorrelation for all infection markers.

9. Line 202: “locations of significant clusters”. Whether the data for this analysis is positivity at household level? What does locations refer to? Is it location of households? Please clarify.

**Response:** “Locations of significant clusters” refer to clusters of household locations for Getis-Ord Gi* statistic but a window in SaTScan analysis. We have removed locations from the sentence.

**Page 10, line numbers 223-225:** Two methods of *local* spatial analysis, the Kulldorff’s scan statistic and the Getis-Ord Gi* statistic, were used to identify locations of significant clusters and hotspots of Ag, Mf, and each Ab.

10. Lines 249 & 261 -271: The 2016 community survey include only 2507 persons (including 11 invalid FTS tests) from 711 households (hh) in 30 PSUs (please see Shheel et al.2018). Whereas in the present study the corresponding values were given as 2671 participants from 750 hhs (730 unique locations) in 32 PSUs in 30 villages. The values differ from the original study results. Similarly, the infection results presented in Lines 261-271 are different from that presented in the original study. Please clarify.
    **Response:** The numbers differ between the two studies because Sheel et al only reported results of the randomly selected PSUs. The current study also includes two additional purposively selected PSUs. The details of the additional PSUs have been described in the Methods and previously reported by Lau *et al*. We also updated the Result section to make it clearer to the readers.

**Pages 6-7, line numbers 143-148:** Thirty PSUs (from 28 villages) were randomly selected from a total of 70 villages; results of LF seroprevalence from these PSUs have previously been described by Sheel *et al* [21] and Lau *et al* [19]. In addition, two villages (Fagali’i and Futiga) that were previously identified as hotspots in 2010, and confirmed in 2014, were purposively sampled as additional PSUs and results reported by *Lau et al* [19]. This study includes results from 750 households in all 32 PSUs (across 30 villages).

**Page 12, line number 276:** The 2016 household survey included 2710 participants from 32 PSUs in 30 villages [20, 21].

11. Please cite PLOS NTD 2020, instead of ‘medRxIV’ for ref. 20.
    - Delete the repeat citation ref. 34, which is same as 20.
    - Please check the citation 34: should it be 19?

**Response:** We have corrected the citations in the revised manuscript.

12. Lines 290-291: How was the statistical significance of spatial-dependency determined? Please mention the statistical test used under Methods and its results in the Table or text.

**Response:** We have revised the methods to include significance testing of spatial dependency.

**Page 9, line numbers 196-203:** A semivariogram is defined by three parameters: the sill- the semivariance at which the variogram plateaus (indicative of statistical significance vs no plateauing if not significant); the nugget- the value at which semivariance intercepts the y-axis (represents spatial variability or measurement error); and the range- the distance between the y-axis and point at which the sill first flattens out (represents the size of geographical clusters). Partial sill is the sill minus the nugget. The proportion of the variation attributed to spatial structure (geographical proximity) can be calculated by dividing the partial sill by the sum of partial sill and nugget.

13. Table 2 may be deleted, as the semivariogram (Fig. 7) describes the spatial dependency. All relevant data are already given in the text.

**Response:** The Table 2 has more information than presented in Fig. 7 so we prefer to keep it as presented in the current form.

14. Table 3: Title is not clear and should also include “Mf”. Retitle it as “Summary statistics from SaTScan^TM^ using Bernoulli model for identifying significant clusters of microfilaria, antigen and antibody positives”.

**Response:** The title of Table 3 has been revised as suggested.

15. Please explain how the RRs inside the window were calculated for each significant cluster. More specifically, what is the reference group for calculating the RR.

**Response:** The details on the calculation of RR has been described in the Methods section.

**Page 10, line numbers 232-236:** SaTScan takes into account the observed number of positive and negative individuals inside and outside the windows, calculates the relative risk (RR) of positive cases inside each window, and reports locations of windows (clusters) where there is a statistically significantly higher proportion of positive cases within the window [39, 40].

16. Fig 8. The prevalence of Ag and Mf within all significant clusters are higher than that of antibody clusters. This is somewhat unexpected. Please explain.

**Response:** Figure 8 actually shows that the prevalence of Ag and Mf are LOWER than the prevalence of Ab in each cluster. In the Ag cluster, SaTScan was focused on analysing Ag results, so it was not unexpected that Ag prevalence was very high in this cluster. Similarly, Mf prevalence was very high in the Mf cluster. The important finding here is that even in Ab clusters, there was high prevalence of Ag and Mf compared to the overall prevalence in the study population (panel a), highlighting the potential for using Ab clusters to identify areas with high Ag and Mf prevalence.

17. Lines 366-381: The semi-variogram analyses measures spatial auto-correlation of the outcome interest for all pairs of observations within each lag. The estimated cluster sizes are expected to be independent of sampling methods, provided the samples have spatial representation in the study area. Therefore, the larger cluster sizes for Ag and smaller for Wb123 estimated in 2010 study compared to the present study could not be attributed to difference in sampling methods. But the temporal changes in Ag-prevalence could have impacted the cluster sizes.

    Another explanation could be that the tests used for detecting antigen were different (og4C3 vs FTS): FTS is more sensitive than Og4C3. Therefore, the above explanation may be applicable to Ag but not for Wb123 Ab as it is expected to be for a longer period even in the absence of resurgence and hence though an increase in Ab prevalence is expected if there is any resurgence but not a reduction. The reason for smaller cluster size for Wb123 in the 2010 survey compared to that in 2016 is difficult to explain. I would suggest the authors to revise the text in light of the above, if agreeable or else need clarification.

**Response:** The 2010 study used a spatial sampling strategy that included all villages, while the 2016 study was a cluster survey and did not include all villages. The lack of spatial representation in some areas in 2016 may therefore have contributed to the difference in cluster sizes. We are not aware of the evidence that FTS is more sensitive than Og4C3. Even if it were, given the low Ag prevalence, we do not expect that any small differences in sensitivity between tests would have any significant impact on cluster size. Regarding Wb123 Ab, this paragraph referred to a reduction in cluster size, but not a reduction in Ab prevalence. We have proposed a possible explanation for the differences in cluster sizes between the two studies (different sampling strategies and significant increase in prevalence), but we do not have any clear answers at this stage for the reduction in cluster size for Wb123 Ab.

We have amended the manuscript to read:

**Page 17, line numbers 400-402:** Different sampling methods were used: in 2010 (spatial sampling strategy that included all villages) and 2016 (village cluster survey of selected villages).

18. Line 470: references to earlier related work on spatial risk map may be added (e.g. Sabesan et al. (2013). Vector borne and zoonotic diseases 13(9). DOI: 10.1089/vbz.2012.1238).

**Response:** The suggested citation has been added to the revised manuscript.

19. Discussion is too lengthy to read, the authors may consider shortening the discussion.

**Response:** We have shortened the manuscript by removal of repetitive parts of several paragraphs as well as extraneous words.
