# Supplementary material for: Lymphatic filariasis in 2016 in American Samoa: Identifying clustering and hotspots using non-spatial and three spatial analytical methods
Source: PLoS Negl Trop Dis. 2022 Mar 28;16(3):e0010262. doi: 10.1371/journal.pntd.0010262 (PMC8989349; doi:10.1371/journal.pntd.0010262)
Supplement: Attachment — Submitted filename: Comments on PNTD-D-21-00923_R1_ASV.docx [file pntd.0010262.s006.docx]

**Comments on the revised manuscript (PNTD-D-21-00923_R1)(entitled ”Lymphatic filariasis in 2016 in American Samoa: Identifying clustering and hotspots using non-spatial and three spatial analytical methods” no: by Wangdi et al**

Thanks the authors for their point by point response to all the queries raised with regard to the manuscript. They have taken sincere efforts to address most queries with adequate valid explanations and have revised the manuscript accordingly. Congrats to the team. Now the manuscript seems complete with all necessary information embedded in the appropriate places.

However, there are few queries which the authors can still address to make this manuscript a wholesome paper with regard to the application of spatial clustering in terms of different indicators in understanding the dynamics of infection during the last phases of elimination.

1. The study aims to identify clustering and hotspots of LF through different infection indicators, an important aspect as we near elimination. All these analysis based on the observed data do identify clusters. After we achieve elimination, it is expected the post elimination surveillance will be on and the programme may start targeting case by case. In such a situation how helpful will these analytical methods be particularly when the infection levels will be at ultra low level.
2. The authors have mentioned that the analysis has been made only at household level – in page 14 lines 332-334, clustering is mentioned in terms of two areas as well indicating clustering at the level of sites as well. The authors could discuss on how the site level clustering will be more beneficial while targeting for interventions.
3. In the comment 30, a table can still be given so shown which sites exhibit clusters at HH level. This would be easier to visualize if there is a possibility of clustering at site level too.
4. The definition of resurgence given by authors says “a significant increase in infection level” – what do they mean by this significant increase – any threshold value. Otherwise the resurgence sounds arbitrary. May be it needs to be worded carefully
5. This is similar to (1). The response to the query on the use of identifying clusters is very ambiguous. One would expect that with the pressure of MDA rounds it is possible that we may finally reach a situation where there will be occurrence of cases here and there and no more cluster of cases, particularly in a post –elimination setting. How useful will these methods be in such a situation? Ideally these methods are more appropriate to use during MDA or during the post MDA phase prior to validation of elimination rather than post elimination phase.
6. In the response to the query 27, the authors have mentioned “Antibodies are potentially useful for identifying clusters of MF /Ag positive people. “. If so, suppose a cluster occurs in terms of antibodies, can one be sure that this area requires MDA? It is seen in many studies that presence of Ab does not necessarily mean presence of Ag or Mf.
7. Can the authors also suggest which Ab assay (Bm1 or Bm33 or Wb123) can be recommended to be used along with Ag to detect resurgence, accounting for the knowledge what each of these Ab assays detects
8. In the query 33, the authors are again repeating what is already known. There is no information on how these are useful in terms of the LF elimination game plan. Few words in this direction would strengthen the manuscript
