# Supplementary material for: Lymphatic filariasis in 2016 in American Samoa: Identifying clustering and hotspots using non-spatial and three spatial analytical methods
Source: PLoS Negl Trop Dis. 2022 Mar 28;16(3):e0010262. doi: 10.1371/journal.pntd.0010262 (PMC8989349; doi:10.1371/journal.pntd.0010262)
Supplement: Attachment — Submitted filename: Comments on the revised manuscript PNTD-D-21-00923_R1.docx [file pntd.0010262.s007.docx]

**Comments on the revised manuscript PNTD-D-21-00923_R1**

The authors have revised the manuscript addressing most of my concerns satisfactorily. I congratulate the authors for demonstrating the potential usefulness of different non-spatial and spatial methods for characterizing LF clusters and hotspots based on different LF indicators. I have a few more questions which I believe is important before the ms is accepted for publication.

1. **Line 110-114:** As the target populations are different for the community survey (> years) and TAS (6-7 yo), it is not clear, whether the community survey is more sensitive than TAS in detecting more ag-positive children (6-7 yo)? The words “for identifying Ag-positive people compared with the school-based TAS of 6-7-year-old children, and was able” may be deleted.
2. **Line 197-98, 318-19 & 394-308:** The response is not satisfactory. The authors have qualitatively measured spatial dependency but state “statistically significant spatial dependency”. What percentage of variance (partial sill/sill = 1-nugget/sill) due to spatial dependence (Table 2) could be indicative of statistically significant spatial dependency? The ratio of nugget to sill indicates the spatial dependency of variables. There are three classiﬁcations used for model explanation: If the ratio is less than 25 %, it shows strong spatial dependence; if the ratio is in between 25 and 75 %, it indicates moderate spatial dependence; and if the ratio is more than 75 %, it represents weak spatial dependence. I believe the % of variance reported in Table 2 due to spatial dependence must be 86% (nugget/sill), 74%, 63%, 79% and 60% for Ag, Mf, Wb123, Bm14, and Bm33 respectively, suggesting moderate spatial dependence for Mf, Wb123 and Bm33 and weak dependence for Ag and Bm14. Accordingly, the authors need to revise Methods for inclusion of the above, Table 2, the relevant Results and discussion in Line 394-408, if the authors agree or else please clarify.

**Line 201-03:** Please revising the text as “……by dividing the nugget by sill” instead of “….by dividing the partial sill by the sum of partial sill and nugget”.

**In Figure 7,** what does the solid line refer to? Whether the solid line is based on fitting any semivariogram model to the semi variance. Please clarify.

Further, (optionally) I would suggest to use the Mantel test for checking the spatial dependency using the package “vegan” in R. The Mantel test can be carried out by calling the “mantel function”. By default, the mantel function uses 1000 permutations (default) for the permutation test and provide a crude estimate of statistical significance. A Mantel test provides evidence for a spatial association but is incapable of determining the nature of that association.

Please see for more details: Manly, Bryan F. J. 2001. Statistics for Environmental Science and Management. Chapman & Hall/CRC Press: Boca Raton, FL.

3. **Line 220-21:** Thanks for including the results of Moran’s I in the Supplementary Table 1. However, I do not understand why the authors just mentioned in the Methods section without even referring them in the Results. I would suggest the authors to delete “(Supplementary Table 1)” here and refer the Supplementary Table 1 in the Results section and interpret and discuss connecting the results of other spatial methods.
4. **Line 227-28:** “relative magnitude between observations”. Relative magnitude of what? between observation means “between households / hotpots.” Please make it clear.
5. **Line 296:** replace “where” with “for whom”
6. **Line 297:** replace “n=34” with “no. of Mf-positves=34”
7. **Lines 446-448:** Please reword the sentence as “The absence of Bm14 and Bm33 Ab hotspots in this area may indicate that ~~more recent~~  the transmission is more recent than that in clusters 2 and 3 because these antibodies persist for years.
8. The authors can consider deleting the word “three” in the title of the manuscript.

**********
