# Supplementary material for: Lymphatic filariasis in 2016 in American Samoa: Identifying clustering and hotspots using non-spatial and three spatial analytical methods
Source: PLoS Negl Trop Dis. 2022 Mar 28;16(3):e0010262. doi: 10.1371/journal.pntd.0010262 (PMC8989349; doi:10.1371/journal.pntd.0010262)
Supplement: Attachment — Submitted filename: Reviewers_comments_PNTD-D-21-00923_R1_final.docx [file pntd.0010262.s008.docx]

12 January 2022

Editor-in-chief

PLoS NTD

Ref: Submission of revised manuscript no PNTD-D-21-00923R1.

To Editor-in-Chief,

On behalf of my co-authors, I would like to submit the revised manuscript “Lymphatic filariasis in 2016 in American Samoa: Identifying clustering and hotspots using non-spatial and three spatial analytical methods”.

All the reviewer’s comments have been addressed and responses have been outlined in the attached Responses to Reviewer's document.

Thank you for your consideration.

Sincerely,

Dr Kinley Wangdi

Corresponding author

**Reviewer's Responses to Questions**

**Key Review Criteria Required for Acceptance?**

**Methods**
-Are the objectives of the study clearly articulated with a clear testable hypothesis stated?
-Is the study design appropriate to address the stated objectives?
-Is the population clearly described and appropriate for the hypothesis being tested?
-Is the sample size sufficient to ensure adequate power to address the hypothesis being tested?
-Were correct statistical analysis used to support conclusions?
-Are there concerns about ethical or regulatory requirements being met?

Reviewer #1: I think the addition of the 'infection marker' section is a very helpful addition for readers.
**Response:** Thanks.

Reviewer #2: Yes
**Response:** Thanks.

Reviewer #3: Yes to all, except a methodological suggestion to test the significance of "spatial dependency" Please see attachment for details
**Response:** We have addressed this comment under specific responses.

**Results**
-Does the analysis presented match the analysis plan?
-Are the results clearly and completely presented?
-Are the figures (Tables, Images) of sufficient quality for clarity?

Reviewer #1: Happy to see the addition of p-values in the results.
**Response:** Thanks for your comments.

Reviewer #2: Yes, adding one more table could be useful.
As suggested in the earlier comments, fitting distributions (Poisson/Negative Binomial) to the no. of positives at the household level for different indicators also is a non-spatial analysis to indicate if the distribution of cases are random or clustered.
**Response:** As mentioned in our previous response, we did not fit the distributions of the frequency of number infected persons per households for different indicators. We don’t believe that this analysis will be useful or accurate because the number of participants varied widely between households (mean of 6, range 1-25), and not all household members were tested. Instead, we used mixed effects logistic regression models to calculate intra-cluster correlation (ICC) for each infection marker. The results are presented in Table 1, showing variation in ICC between infection markers, and more intense clustering (higher ICC) at household level than village level for all infection markers.

Reviewer #3: Yes to all with minor revisions in the Results based on the suggested method (indicated above) in Table 2 and Discussion.
**Response:** We have addressed this comment under specific responses.

**Conclusions**
-Are the conclusions supported by the data presented?
-Are the limitations of analysis clearly described?
-Do the authors discuss how these data can be helpful to advance our understanding of the topic under study?

-Is public health relevance addressed?

**Reviewer #1:** none

**Reviewer #2**: The conclusion may be strengthened in terms of how these analytical methods would be helpful in post elimination phase where the cases would have drastically come down and may not exhibit any clusters. Also on the indicator that could be used not to miss out any cluster during the post validation phase.

**Response:** We have addressed this under specific responses.

Reviewer #3: Yes to all, but depending on the revisions based on the spatial dependency analysis suggested above.

**Response:** We have addressed this under specific responses.

**Editorial and Data Presentation Modifications?**
Use this section for editorial suggestions as well as relatively minor modifications of existing data that would enhance clarity. If the only modifications needed are minor and/or editorial, you may wish to recommend “Minor Revision” or “Accept”.

Reviewer #1: none
Reviewer #2: None
Reviewer #3: Please see attachment for minor corrections

**Response:** Thanks to all the reviewers. We have made point-by-point responses to each reviewer’s comments.

**Summary and General Comments**
Use this section to provide overall comments, discuss strengths/weaknesses of the study, novelty, significance, general execution and scholarship. You may also include additional comments for the author, including concerns about dual publication, research ethics, or publication ethics. If requesting major revision, please articulate the new experiments that are needed.

Reviewer #1: The authors addressed my initial minor feedback from the first review. I think the manuscript is strong and adds to ongoing work on geospatial analysis and clustering of infection, both of which are important as LF elimination programs are reaching the last mile.
**Response:** Thanks.

Reviewer #2: This manuscript is important particularly as it compares different spatial clustering techniques.

**Response:** Thanks for your comments.

Reviewer #3: The authors have revised the manuscript addressing most of my concerns satisfactorily. I congratulate the authors for demonstrating the potential usefulness of different non-spatial and spatial methods for characterizing LF clusters and hotspots based on different LF indicators. I have a few more questions which I believe is important before the ms is accepted for publication.

**Response:** We have made point by point responses in the individual response document.

**Comments on the revised manuscript PNTD-D-21-00923_R1**

The authors have revised the manuscript addressing most of my concerns satisfactorily. I congratulate the authors for demonstrating the potential usefulness of different non-spatial and spatial methods for characterizing LF clusters and hotspots based on different LF indicators. I have a few more questions which I believe is important before the ms is accepted for publication.

1. **Line 110-114:** As the target populations are different for the community survey (> years) and TAS (6-7 yo), it is not clear, whether the community survey is more sensitive than TAS in detecting more ag-positive children (6-7 yo)? The words “for identifying Ag-positive people compared with the school-based TAS of 6-7-year-old children, and was able” may be deleted.

**Response:** Our previous paper (Sheel et al, PLOS NTDs 2016) provides detailed comparison of the two surveys and concluded that TAS of 6-7 year-old children was less sensitive than community survey of older age groups for identifying foci of ongoing transmission. We have reworded the sentence to the following:

**Page 5, line numbers 111-115:** The study demonstrated that the community-based survey of older age groups (≥8 years) was more sensitive than TAS of 6-7 year-old children for identifying signals of ongoing transmission, including hotspots identified in 2010 and 2014 studies: Fagali’i village in the far north-west of Tutuila island and a group of three villages (Ili’Ili, Vaitogi and Futiga) on the south coast [20, 21].

2. **Line 197-98, 318-19 & 394-308:** The response is not satisfactory. The authors have qualitatively measured spatial dependency but state “statistically significant spatial dependency”. What percentage of variance (partial sill/sill = 1-nugget/sill) due to spatial dependence (Table 2) could be indicative of statistically significant spatial dependency? The ratio of nugget to sill indicates the spatial dependency of variables. There are three classiﬁcations used for model explanation: If the ratio is less than 25 %, it shows strong spatial dependence; if the ratio is in between 25 and 75 %, it indicates moderate spatial dependence; and if the ratio is more than 75 %, it represents weak spatial dependence. I believe the % of variance reported in Table 2 due to spatial dependence must be 86% (nugget/sill), 74%, 63%, 79% and 60% for Ag, Mf, Wb123, Bm14, and Bm33 respectively, suggesting moderate spatial dependence for Mf, Wb123 and Bm33 and weak dependence for Ag and Bm14. Accordingly, the authors need to revise Methods for inclusion of the above, Table 2, the relevant Results and discussion in Line 394-408, if the authors agree or else please clarify.

**Response:** Thanks for the suggestions.

- We calculated the percentage of variance due to spatial dependence by dividing the partial sill by the sum of partial sill and nugget (references: Chiles, J.-P., & Delfiner, P. (2009). Geostatistics: Modeling spatial uncertainty. John Wiley & Sons. Cressie, N. (2015). Statistics for spatial data. John Wiley & Sons). This is mathematically equivalent to the formula provided by the reviewer above (partial sill/sill = 1-nugget/sill) since partial sill = sill-nugget. The formula is often expressed as partial sill / (partial sill+nugget) because these are the parameters reported by the geoR package.
- Statistical significance of spatial dependence is indicated by plateauing of the semivariogram, vs no plateauing if not significant. Please see lines 203-205 in Methods.
- As suggested by the reviewer, we have included the results for nugget/sill to Table 2 as an indicator of the strength of spatial dependence. And provided explanation in Methods (lines 210 and 212) and results (lines 336-337): The nugget to sill ratio indicated moderate spatial dependence for Mf, Wb123 Ab and Bm33 Ab, and weak dependence for Ag and Bm14 (**Table 2**).

**Page 9, line numbers 201-205:** The proportion of the variation attributed to spatial structure (geographical proximity) can be calculated by dividing the partial sill by the sum of partial sill and nugget (equal to dividing nugget by sill) [38, 39]. The ratio of nugget to sill provides a measure of the strength of spatial dependence, where ratios of <25%, 25-75%, and >75% indicate strong, moderate, and weak dependence, respectively.

**Page 14, line 326-327: The** nugget to sill ratio indicated moderate spatial dependence for Mf, Wb123 Ab and Bm33 Ab, and weak dependence for Ag and Bm14 (**Table 2**).

3. **Line 201-03:** Please revising the text as “……by dividing the nugget by sill” instead of “….by dividing the partial sill by the sum of partial sill and nugget”.

**Response:** The equation that we used is partial sill / (partial sill + nugget). As explained above, this is the same as nugget / sill. We have added this to the text and included two references: References: Chiles, J.-P., & Delfiner, P. (2009). Geostatistics: Modeling spatial uncertainty. John Wiley & Sons. Cressie, N. (2015). Statistics for spatial data. John Wiley & Sons.

**Page 9, line numbers 201-203:** The proportion of the variation attributed to spatial structure (geographical proximity) can be calculated by dividing the partial sill by the sum of partial sill and nugget (equal to dividing nugget by sill) [38, 39].

4. **In Figure 7,** what does the solid line refer to? Whether the solid line is based on fitting any semivariogram model to the semi variance. Please clarify.

Further, (optionally) I would suggest to use the Mantel test for checking the spatial dependency using the package “vegan” in R. The Mantel test can be carried out by calling the “mantel function”. By default, the mantel function uses 1000 permutations (default) for the permutation test and provide a crude estimate of statistical significance. A Mantel test provides evidence for a spatial association but is incapable of determining the nature of that association.

Please see for more details: Manly, Bryan F. J. 2001. Statistics for Environmental Science and Management. Chapman & Hall/CRC Press: Boca Raton, FL.

**Response:** The solid lines in the Fig 7 is the spatial dependence of positive results at the household level modelled using semivariograms.

**Page 9, line numbers 206-208:** For each infection marker, spatial dependence of positive results at the household level was investigated using semivariograms in the statistical software R, using the variog function in geoR package version 2.14.1 (The R Foundation for Statistical Computing).

Thanks for the suggestion to optionally explore the Mantel test – this appears to be most useful for analysing correlation between two datasets, which is not directly applicable to our study.

5. **Line 220-21:** Thanks for including the results of Moran’s I in the Supplementary Table 1. However, I do not understand why the authors just mentioned in the Methods section without even referring them in the Results. I would suggest the authors to delete “(Supplementary Table 1)” here and refer the Supplementary Table 1 in the Results section and interpret and discuss connecting the results of other spatial methods.

**Response:** We have incorporated the suggested changes in the revised manuscript.

**Page 14, line number 328:** Moran’s I Statistics showed significant global clustering for all seromarkers (**Supplementary Table 3**).

6. **Line 227-28:** “relative magnitude between observations”. Relative magnitude of what? between observation means “between households / hotpots.” Please make it clear.

**Response:** We have revised the sentence as follows (further details about Kulldorff’s scan statistics are provided further down in the section in lines 229-232).

**Page 10, line numbers 228-231:** The Kulldorff’s scan statistic was used to determine the geographic distribution of clusters of high prevalence of infection markers, while Getis-Ord Gi* statistic was used to test the statistical significance of hotspots and to determine the spatial dependence between observations (see below).

7. **Line 296:** replace “where” with “for whom”
8. **Line 297:** replace “n=34” with “no. of Mf-positves=34”

**Response:** We have made the suggested changes.

**Page 13, line numbers 298-301:** Using a denominator of 2650 (2671 minus 21 Ag-positive participants for whom slide results were not available), Mf prevalence was 1.3% (no. of Mf positives=34). Crude Mf, Ag, and Ab (Bm14, Wb123, and Bm33) prevalence by age and gender are presented in **Fig 1**.

9. **Lines 446-448:** Please reword the sentence as “The absence of Bm14 and Bm33 Ab hotspots in this area may indicate that the transmission is more recent than that in clusters 2 and 3 because these antibodies persist for years.

**Response:** We have made the suggested changes as follows.

**Page 19, line numbers 452-454:** The absence of Bm14 and Bm33 Ab hotspots in this area may indicate that transmission was more recent than those in clusters 2 and 3 because these antibodies persist for years.

10. The authors can consider deleting the word “three” in the title of the manuscript.

**Response:** We believe that it is useful to leave the word “three” in the title to highlight that multiple spatial methods were used.

**Comments on the revised manuscript (PNTD-D-21-00923_R1) (entitled ”Lymphatic filariasis in 2016 in American Samoa: Identifying clustering and hotspots using non-spatial and three spatial analytical methods” no: by Wangdi et al**

Thanks the authors for their point by point response to all the queries raised with regard to the manuscript. They have taken sincere efforts to address most queries with adequate valid explanations and have revised the manuscript accordingly. Congrats to the team. Now the manuscript seems complete with all necessary information embedded in the appropriate places.

However, there are few queries which the authors can still address to make this manuscript a wholesome paper with regard to the application of spatial clustering in terms of different indicators in understanding the dynamics of infection during the last phases of elimination.

1. The study aims to identify clustering and hotspots of LF through different infection indicators, an important aspect as we near elimination. All these analyses based on the observed data do identify clusters. After we achieve elimination, it is expected the post elimination surveillance will be on and the programme may start targeting case by case. In such a situation how helpful will these analytical methods be particularly when the infection levels will be at ultra low level.

**Response:** Thanks for the question. Our previous studies in American Samoa and Samoa identified significant household level clustering, so spatial clustering might actually become more intense as infection reaches very low levels, i.e. any residual infections are less likely to be randomly distributed over space and more likely to be focal. The use of cluster analysis may therefore be particularly useful for delineating the areas where ongoing transmission is most likely, and conducting targeted testing and treating of the population within these areas. Further studies will be required to determine the most effective and efficient approach for post elimination surveillance in different settings.

We have added the following text in **pages 19-20, lines 478 to 487** to address a number of the concerns raised here:
The programmatic value of the results will vary depending on the stage of elimination and also differ between countries depending on programme and history of endemicity. It is likely that spatial clustering will become more intense as infection reaches very low levels, so cluster analysis may help to delineate and prioritise areas of ongoing transmission before infection can spread and lead to more widespread resurgence. Given the greater sensitivity of Abs for cluster identification compared to Ag and Mf, Ab markers could be an additional tool for identifying areas for test and treat programmes. However, further studies of the relationships between Abs, Ag and Mf over space and time are needed before recommendations about more sensitive and specific diagnostic strategies or markers can be made.

2. The authors have mentioned that the analysis has been made only at household level – in page 14 lines 332-334, clustering is mentioned in terms of two areas as well indicating clustering at the level of sites as well. The authors could discuss on how the site level clustering will be more beneficial while targeting for interventions.

**Response:** Spatial analysis was done using household-level data (i.e. point data). The description in lines 333 and 334 referred to the villages where clusters were identified, but we did not conduct spatial analysis at the village level. For larger countries, similar analyses could potentially be conducted at the village level, ideally using household-level point data if possible. If household level data were not available, village level data could be mapped to a location in the village (e.g. the centroid or geographic centre of the village) and used to conduct spatial analyses similar to what we have done in this paper. We have expanded the Discussion about how identification of clusters could be useful for targeting interventions.

3. In the comment 30, a table can still be given so shown which sites exhibit clusters at HH level. This would be easier to visualize if there is a possibility of clustering at site level too.

**Response:** Cluster analysis at the household level (ICC) was conducted using data in the whole study area. The ICCs provide a global measure of clustering at the household and village levels (Table 1) but do not provide information about household level clustering at specific villages.

4. The definition of resurgence given by authors says “a significant increase in infection level” – what do they mean by this significant increase – any threshold value. Otherwise the resurgence sounds arbitrary. May be it needs to be worded carefully.

**Response:** In our previous response to reviewers, we commented that “Resurgence is a commonly used term to indicate significant increase in infection prevalence to levels above target thresholds, indicating increase in transmission. (e.g. Prada et al. <https://academic.oup.com/jid/article/221/Supplement_5/S503/5680710?login=true>). We have added this definition to the Introduction.

**Page 5, line numbers 107-109:** The term “resurgence” was used to indicate significant increase in infection prevalence to levels above target thresholds.

5. This is similar to (1). The response to the query on the use of identifying clusters is very ambiguous. One would expect that with the pressure of MDA rounds it is possible that we may finally reach a situation where there will be occurrence of cases here and there and no more cluster of cases, particularly in a post –elimination setting. How useful will these methods be in such a situation? Ideally these methods are more appropriate to use during MDA or during the post MDA phase prior to validation of elimination rather than post elimination phase.

**Response:** Please see our response above under (1). We agree that the value of spatial analyses will vary depending on the stage of elimination, and will also likely differ between countries. We have expanded on this point in the Discussion.

6. In the response to the query 27, the authors have mentioned “Antibodies are potentially useful for identifying clusters of MF /Ag positive people. “. If so, suppose a cluster occurs in terms of antibodies, can one be sure that this area requires MDA? It is seen in many studies that presence of Ab does not necessarily mean presence of Ag or Mf.

**Response:** We agree that the presence of an Ab cluster does not necessarily mean the presence of Ag or Mf positive people. The need for more sensitive and specific diagnostics is one of the key challenges for LF elimination programs. Our study is a preliminary investigation into the potential use of antibodies, including their spatial distribution, as an additional tool for identifying Ag or Mf positive people. Currently, there are no official guidelines for the use of antibodies for informing any programmatic activities, including MDA.

7. Can the authors also suggest which Ab assay (Bm1 or Bm33 or Wb123) can be recommended to be used along with Ag to detect resurgence, accounting for the knowledge what each of these Ab assays detects.

**Response:** There is currently insufficient evidence for such specific recommendations. Further studies like our current study are needed to improve understanding of the relationships between antibodies, Ag, and Mf, and generate evidence for guiding future recommendations.

8. In the query 33, the authors are again repeating what is already known. There is no information on how these are useful in terms of the LF elimination game plan. Few words in this direction would strengthen the manuscript.

**Response:** Our responses to query 33 (included below for ease of reference) highlighted how our study contributes new knowledge about spatial analyses and antibodies – these points were not already known. As above, there is currently insufficient evidence for making specific recommendations for the use of antibodies in LF surveillance.

*(Q33 from previous revision: The manuscript should indicate what additional information has been provided by this analysis and how this would be useful in terms of achieving LF elimination goal.*

***Response:*** *Our manuscript provided multiple advances in knowledge that are relevant to LF elimination:*

- *We explored the use of spatial and non-spatial methods for measuring clustering at household and village levels, and for identifying locations of clusters. Non-spatial method (ICC) provides a general measure of the level of clustering at household and village levels across the whole study area, while spatial methods show location specific information.*
- *We confirmed that these methods identified known clusters, as well as new clusters*
- *We have provided evidence for the potential utility of Abs in LF surveillance, including the use of Ab clusters to identify Ag and Mf clusters (line numbers 436-438).)*
